# Main Pathological Changes of Benign Ureteral Strictures

**DOI:** 10.3389/fmed.2022.916145

**Published:** 2022-07-07

**Authors:** Jiang Tan, Zhuoyuan Yu, Xinyi Ling, Guoping Qiu, Xin Yang, Yi Tang, Dong Yang, Mei Yang, Fei Gao

**Affiliations:** ^1^Department of Anatomy, Institute of Neuroscience, College of Basic Medicine, Chongqing Medical University, Chongqing, China; ^2^Department of Urology, The First Affiliated Hospital of Chongqing Medical University, Chongqing, China; ^3^Department of Pathology, The First Affiliated Hospital of Chongqing Medical University, Chongqing, China; ^4^Molecular Medicine Diagnostic and Testing Center, Chongqing Medical University, Chongqing, China; ^5^Department of Radiology, The First Affiliated Hospital of Chongqing Medical University, Chongqing, China

**Keywords:** ureteral stricture, histopathology, fibroblasts, leukocytes, collagen, hydronephrosis

## Abstract

**Objective:**

To identify the pathological classification of benign ureteral strictures according to the histological features and explore the relationship between various pathological types and inflammatory cells, fibroblasts, and collagen.

**Patients and Methods:**

Thirty one specimens from patients diagnosed with ureteral strictures between 2013 and 2021 were included and classified according to the histopathological characteristics. The number of fibroblasts and inflammatory cells was counted, and the proportion of type I and type III collagen in ureteral stricture tissues was detected by picrosirius red staining.

**Results:**

We identified three types of benign ureteral strictures in 31 specimens: inflammatory cell infiltration (*n* = 10, 32%), fibroplasia (*n* = 14, 45%), and hyalinization (*n* = 7, 23%), with significant differences in obstruction history and hydronephrosis grades among the three types. The number of inflammatory cells (lymphocytes, neutrophils and eosinophils) was significantly lower in hyalinization ureteral strictures than in the other two types (*p* < 0.05). The number of foreign-body giant cells associated with foreign-body reactions increased significantly in suture-induced ureteral strictures (*p* < 0.05). Fibroplasia type had the largest number of fibroblasts, whereas the other two types had smaller numbers. The results of type I and III collagen analysis showed that type I and III collagen were the most abundant in hyalinization among all ureteral stricture types (*p* < 0.05). Compared to ureteral strictures, the content of type I and III collagen in atresia increased significantly (*p* < 0.05).

**Conclusion:**

Common pathological types of benign ureteral strictures include inflammatory cell infiltration, fibroplasia, and hyalinization. Changes in type I and III collagen, inflammatory cells, and fibroblasts in different pathological types may be related to the progression of ureteral strictures.

## Introduction

Ureteral strictures are characterized by high incidence and recurrence rate, which result in narrowing of the ureteral lumen, causing upper urinary tract obstruction, hydronephrosis, and even renal failure. The problems of high recurrence of restenosis, unstable curative effect, and inevitable nephrectomy after long-segment ureteral strictures unavoidably persist, making it a challenging disease. One of the reasons making it challenging is that the histopathological characteristics have not been systematically reported before, therefore, studies reporting histopathology are valuable for further research into the molecular mechanisms and clinical management of ureteral strictures.

Stones, injuries, and sutures can lead to strictures of ureters. Inflammation following stones, foreign matter, and injuries, activates fibroblasts to a large extent, which regulates cell proliferation and migration and significantly upregulate collagen production, collagen deposition, and the expression of other extracellular matrix components (1).

Fibroblasts are typically spindle-shaped cells with an oval flat nucleus in the interstitial spaces of organs (2). They are fundamental to the development of scar formation, which is in the scar proliferative phase, whereby fibroblasts start with building up fresh connective tissue by producing and depositing large amounts of collagen (3), which shows great significance in fibrosis diseases.

Several studies have shown that inflammatory cell infiltration is involved in modulating collagen synthesis and fibrotic diseases (4). Regenerating failure has long been associated with scarring or fibrosis, a phenomenon that is the direct result of inflammatory interactions between immune cells and fibroblasts at the site of injury (5). Lymphocytes not only promote the synthesis and metabolism of collagen but also suppress fibroblast proliferation and downregulate type I and III collagen (6).

Meanwhile, the collagen content of tissues related to fibrosis diseases also changed dynamically, which was consistent with the change trend of fibroblasts (7). It has been suggested that the formation, regression, and maturation of collagen can be closely related to fibroblasts (7). Collagen is secreted by fibroblasts, such as type I, II, III, IV and V, in which type I collagen is mainly distributed in the skin, tendon, and other tissues. Type III collagen mostly appears in the skin, intima, and intestines of infants (8). The amount of type III collagen temporarily increases during skin injury and formation of the endodermis (9). It suggests that collagen expression has temporal control in the process of wound healing, and continuous expression of proliferative type III collagen could indicate a change in normal healing results.

In the present study, we firstly propose a commonly applicable pathological partition that includes three pathological types: hyalinization, fibroplasia, and inflammatory cell infiltration. In the relatively mature hyalinization-type ureteral strictures, there was a significant increase in the content of type I and III collagen and a significant decrease in the content of fibroblasts. These findings have significant implications for elucidation of the molecular mechanisms underlying ureteral strictures.

## Patients and Methods

### Patients and Specimens

All 31 specimens from patients diagnosed with ureteral strictures between 2013 and 2021 were included and classified according to their histopathological characteristics.

We included ureteral specimens consistent with ureteral strictures and excluded those that might have been compressed by an adjacent tumor. Detailed inclusion criteria and exclusion criteria are present in Table 1.

**TABLE 1:** Inclusion and exclusion criteria.

Inclusion criteria	Clinical manifestations of upper urinary tract obstruction, preoperative imaging showed hydronephrosis or ureteral stricture, and postoperative pathology confirmed ureteral stricture.
Exclusion criteria	a. Ureteral tumor.
	b. Metastasis of malignant tumors in adjacent organs, such as pelvic tumor, uterine tumor, colon cancer, rectal cancer, and sigmoid colon cancer.

The diagnosis of ureteral strictures relies on symptoms and imaging findings. All patients underwent imaging prior to surgery. Imaging tests included computed tomography (CT), intravenous urography, retrograde pyelogram and anterograde pyelography. Imaging of ureteral strictures, ureteral asteria and hydronephrosis are shown in Supplementary Figure 1.

### Pathology Classifications

To examine the pathological features of the specimens, we first fixed them using 4% paraformaldehyde, then dehydrated them in graded sucrose, embedded them in paraffin, and stained them with hematoxylin and eosin (HE) for general histology.

**FIGURE 1:**
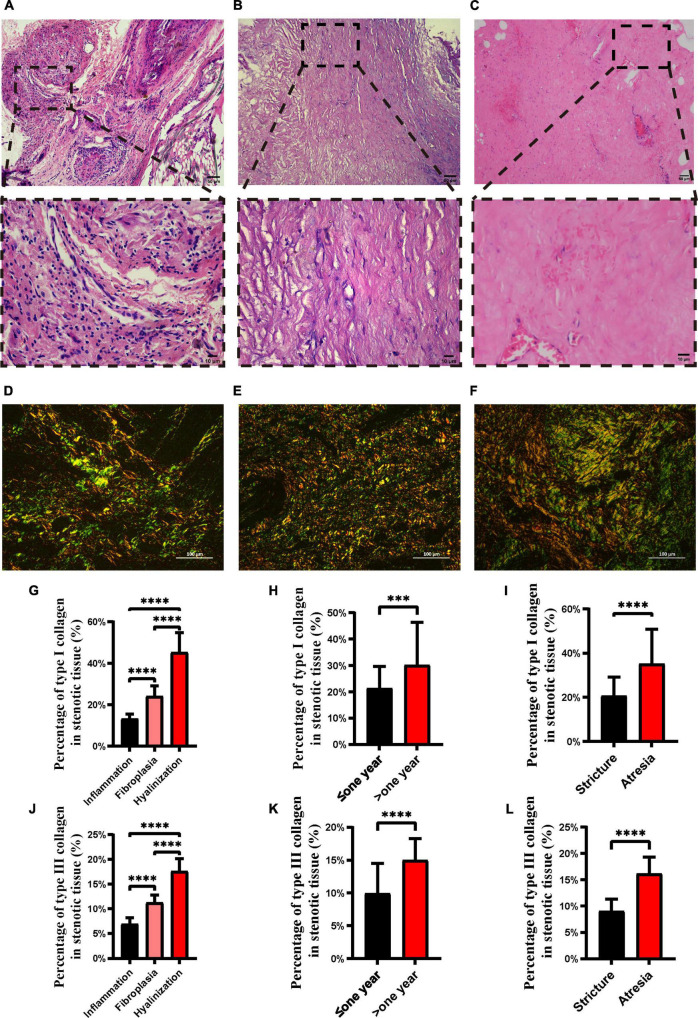
H&E staining and picrosirius red staining of different ureteral stricture histopathology types. H&E staining of inflammation **(A)**, fibroplasia **(B)** and hyalinization **(C)**. Upper panel scale bar = 50 μm; lower panel scale bar = 10 μm. Picrosirius red staining of inflammation **(D)**, fibrous hyperplasia **(E)** and hyalinization **(F)**, where type I collagen is stained red or orange and type III collagen is stained green. Proportion of type I **(G)** and type III **(J)** collagen in the three pathological types of ureteral strictures. Proportion of type I **(H)** and type III **(K)** collagen in the different pathological courses. Proportion of type I **(I)** and type III **(L)** collagen in strictures and atresia. Scale bars, 100 μm (^***^p < 0.001; ^****^p < 0.0001).

According to the general histology of the 31 ureteral strictures samples, three pathological subtypes of ureteral strictures, hyalinization, fibroplasia, and inflammatory cell infiltration were identified by a single, blinded well-experienced pathologist (Figures 1A–C). Specifically, inflammatory cell infiltration is a pathological process in which inflammatory cells accumulate in the lesion, and a large number of inflammatory cell infiltrates can be observed, dominated by lymphocyte and monocyte infiltrates. Fibroplasia is the formation of normal tissue structure replaced by fibrous tissue, which is caused by the proliferation of cells. It is widely accepted that fibroblasts have abundant rough endoplasmic reticulum essential for protein (collagen) synthesis; thus, the cytoplasm is blue on H&E staining (10). Large proliferation of fibroblasts was observed, accompanied by a small infiltration of inflammatory cells, predominantly lymphocytes and monocytes. Hyalinization is a process of transformation of stromal connective tissue into a homogeneous, acellular translucent material (11). In the interstitial tissue, there was uniform, unstructured, translucent protein accumulation, which was eosinophilic and homogeneous in HE staining sections. Marked reduction in fibroblasts and thickening of collagen fibers were observed.

### Picrosirius Red Staining

Specimens were fixed with 4% paraformaldehyde, trimmed, dehydrated, embedded, sliced, dyed, and sealed. The sections were observed under polarized light to evaluate the type, and polarization color of collagen fiber bundles (12) by an Eclipse CI-L microscope (Nikon, Japan), the birefringent collagens were clearly showed up. With a black background, the stained collagen fibers are represented as bright green to red (Figures 1D–F) (13). The target area of ureter tissue was selected under a 200x imaging, then the percentages of type I and type III collagen were analyzed by Image-pro Plus 6.0 analysis after the imaging was completed.

### Fibroblasts and Various Types of Inflammatory Cells Counting

Fibroblasts and various types of inflammatory cells were identified and counted. For counting, typical features of stones, injuries, sutures, inflammatory cell infiltration, fibrous hyperplasia and hyalinization were found in a 400X field, then adjusted to 800X and nine randomly selected fields were used to identify the various cells, and cell counting was conducted.

Previous studies have shown that no single specific cell surface protein marker is expressed on all fibroblasts (14), therefore, instead of performing immunohistochemistry or immunofluorescence, we counted fibroblasts directly based on their morphological characteristics, which are spindle-shaped. Such a counting methods is subject to error, however, our results are still informative.

### Collagen Proportion Calculation

After picrosirius red staining, the proportion of type I and type III collagen was calculated (15). Briefly, the pixels occupied by collagen type I and type III were counted and the respective percentages calculated using Image-pro Plus 6.0 at three randomly selected 200X fields of view.

### Grading of Hydronephrosis

Hydronephrosis is graded using the Society for Fetal Urology (SFU) classification system, and is divided into five grades (16). Grade 0 has an un-dilated pelvis and no change in renal parenchymal thickness. Grade 1 has a mildly dilated pelvis but dilated calyces or parenchymal atrophy; grade 2 has a moderately dilated pelvis, including some calyces; grade 3 has a dilated pelvis, all calyces are uniformly dilated, and the parenchyma is normal; grade 4 has a thinner parenchyma than grade 3, and the rest is the same.

### Statistical Analyses

A Student’s two-tailed unpaired *t*-test, Fisher’s exact test, and one-way analysis of variance (ANOVA) with Tukey’s *post hoc* tests by GraphPad 9.3 (GraphPad Software Inc., United States) and SPSS 26.0 (IBM Inc., United States) was used to perform statistical analysis. Values of *p* < 0.05 were considered statistically significant.

## Results

### Demographic and Clinical Characteristics of Patients

After inclusion and exclusion, we observed and studied a sample of 31 ureteral strictures. Specimens were sampled from 15 females and 16 males who underwent open operation (10 of 31; 32%) or laparoscopic surgery (21 of 31; 68%).

The factors causing strictures included stones (16 of 31; 52%), injuries (10 of 31; 32%) and sutures (5 of 31; 16%). Strictures accounted for 21 of 31 (68%), while atresia accounted for 10 of 31 (32%). At the time of diagnosis, 14 patients had an obstruction history lasting more than 12 months and 17 patients had an obstruction history of less than 12 months. Clinical characteristics are shown in Table 2.

**TABLE 2:** Clinical characteristics of subjects.

Characteristics	Inflammation (*n* = 10)	Fibroplasia (*n* = 14)	Hyalinization (*n* = 7)	*P*-value
Age, years, mean ± *SD*	48.7 ± 9.8	48.1 ± 15.2	45.3 ± 12.9	0.858
**Sex, *n* (%)**				0.240
Male	6 (60.0)	8 (57.1)	2 (28.6)	
Female	4 (40.0)	6 (42.9)	5 (71.4)	
**Factors causing stricture, *n* (%)**				0.567
Stones	4 (40.0)	9 (64.3)	3 (42.9)	
Injuries	3 (30.0)	4 (28.6)	3 (42.9)	
Sutures	3 (30.0)	1 (7.1)	1 (14.3)	
**Type, *n* (%)**				0.122
Ureteral stricture	6 (60.0)	12 (85.7)	3 (42.9)	
Ureteral atresia	4 (40.0)	2 (14.3)	4 (57.1)	
**Obstruction history, months, *n* (%)**				0.041*
≤12	8 (80.0)	8 (57.1)	1 (14.3)	
>12	2 (20.0)	6 (42.9)	6 (85.7)	
**Hydronephrosis grade, *n* (%)**				0.033*
0	0 (0)	0 (0)	0 (0)	
1	5 (50.0)	6 (42.9)	0 (0)	
2	4 (40.0)	2 (14.3)	1 (14.3)	
3	1 (10.0)	5 (35.7)	3 (42.9)	
4	0 (0)	1 (7.1)	3 (42.9)	

P-values were calculated using the Fisher’s exact test or one-way analysis of variance (ANOVA) followed by Tukey’s post-hoc tests, with * indicating statistical significance.

### Three Histopathology Classifications of Ureteral Strictures Are Identified

According to the histological features, the pathologists identified three types of ureteral strictures: inflammatory cell infiltration (Figure 1A), fibroplasia (Figure 1B), and hyalinization (Figure 1C). The pathological characteristics of three types are as follows, accumulation of inflammatory cells, formation of fibrous tissue replacing, and infiltration is the conversion of stromal connective tissue into homogenous and acellular materials, respectively. According to the pathological type, the proportions of inflammatory cell infiltration, fibroplasia and hyalinization were 32% (10 of 31), 45% (14 of 31) and 23% (7 of 31), respectively.

We found that the duration of obstruction was statistically different between pathological types (*p* < 0.05). In addition, hydronephrosis is a common symptom in ureteral strictures, and we found that the grade of hydronephrosis was significantly different among the three types, with the hyalinization type having the highest and inflammatory cell infiltration type having the lowest (*p* < 0.05) (Table 2).

### Inflammatory Counts Vary With Histopathological Typology and Etiology

**FIGURE 2:**
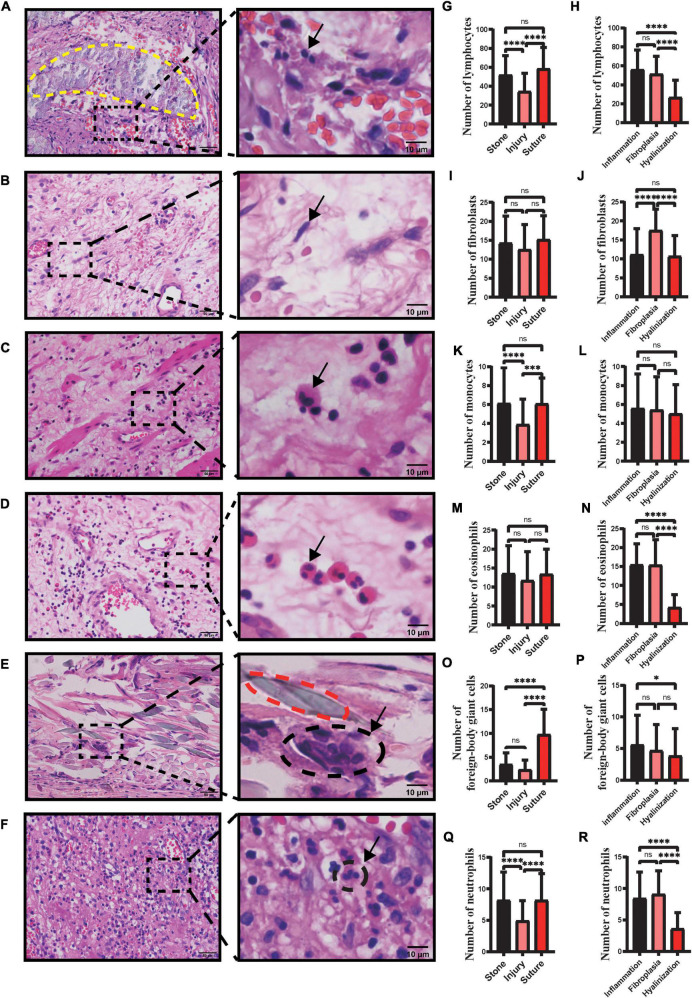
Number of different types of inflammatory cells and fibroblasts in ureteral strictures. HE staining of lymphocytes **(A)**, fibroblasts **(B)**, monocytes **(C)**, eosinophils **(D)**, foreign-body giant cells **(E)** and neutrophils **(F)** at the ureteral stricture site. Arrows point to typical fibroblasts and inflammatory cells. The yellow dashed line **(A)** contains the stone and the red dashed line **(E)** contains the suture. Left column scale bar, 50 μm; right column scale bar, 10 μm. Number of lymphocytes **(G)**, fibroblasts **(I)**, monocytes **(K)**, eosinophils **(M)**, foreign-body giant cells **(O)**, neutrophils **(Q)** in ureteral strictures due to stones, injury, sutures. Number of lymphocytes **(H)**, fibroblasts **(J)**, monocytes **(L)**, eosinophils **(N)**, foreign-body giant cells **(P)** and neutrophils **(R)** in three pathological subtypes: inflammation, fibroplasia and hyalinization (ns, not significant; *p < 0.05; ^***^p < 0.001; ^****^p < 0.0001).

Inflammatory cell infiltration plays an essential role in fibrotic diseases (17). Pulmonary fibrosis is associated with classical inflammatory pathways in which tissue injury evokes inflammatory responses (17). Meanwhile, the inflammation driven by specialized monocyte-derived contributes to Peyronie’s disease (18). In our study, there appeared to be that the profile of the number of the inflammatory cells related to pathological types (Figures 2A–F). We found a significantly reduced proportion of lymphocytes, eosinophils and neutrophils in the hyalinization type compared to fibroplasia and inflammatory cell infiltration (*p* < 0.05) (Figures 2H,N,R). Likewise, the profile of hyalinization showed significantly fewer foreign-body giant cells than the inflammatory cell infiltration type (*p* < 0.05) (Figure 2P). There were no statistically significant differences in the number of monocytes in inflammatory cell infiltration, fibroplasia, and hyalinization (*p* > 0.05), although there was a trend for fewer monocytes to be present in hyalinization.

Injuries, stones, and sutures are infiltrated by diverse inflammatory cells that differentiate into one variety from another. We have sections of stones marked with a yellow dashed line (Figure 2A) and sutures (Figure 2E) marked with a red dashed line. Significantly fewer monocytes were found in the injuries and more foreign-body giant cells were found in the sutures (*p* < 0.05) (Figures 2K,O). Similarly, more lymphocytes and neutrophils were found in sections with stones and sutures, compared to injuries (*p* < 0.05) (Figures 2G,Q). There were no statistically significant differences in the number of eosinophils in injuries, stones, and sutures (*p* > 0.05), although there appeared to be fewer eosinophils in injuries (Figure 2M). In addition, among the factors causing strictures, stones and injuries accounted most of the cases (26 of 31; 84%) (Table 2).

### Fibroblastic Processes Are Active in Fibroplasia Type of Ureteral Strictures and Fibroblasts Are Significantly More Numerous

Fibroblasts participate in collagen synthesis by assembling pre-collagen molecules and releasing them extracellularly *via* exocytosis (19). Resident fibroblasts in the surrounding tissue proliferate and migrate to the wound site, promoting the formation of fibrotic diseases. During fibrogenesis, the number of fibroblasts fluctuates, and an increasing number of fibroblasts suggests active fibrosis (20).

We found that the profile of the number of fibroblasts revealed that the fibroplasia type was ranked the highest among the three types (Figure 2J). However, there were no statistically significant differences in the number of fibroblasts in stones, injuries, and sutures (*p* > 0.05), although the fewest fibroblasts were present in injuries.

### Collagen Type I and III Are Lowest in Inflammatory Cell Infiltration and Highest in Hyalinization

Fibroblasts synthesize proteins, including type I and III collagen, both of which are closely related to strictures. Excessive deposition of collagen family is a hallmark of fibrosis. Type I collagen has load-bearing mechanical properties, and its levels are significantly upregulated during the progression (21). Type III collagen plays an important role in regulating collagen fiber formation, fiber size, and tissue physical properties. A decrease in type III collagen level can lead to excessive proliferation (22).

In the present study, we found that type I and III collagen levels were lowest in inflammatory cell infiltration and highest in hyalinization (*p* < 0.05) (Figures 1G,J). In samples with longer disease duration, type I and type III collagen contents were significantly more abundant (*p* < 0.05) (Figures 1H,K). In addition, a significantly higher proportion of type I and type III collagen was found in atresia than in strictures (*p* < 0.05) (Figures 1I,L).

## Discussion

To the best of our knowledge, there is limited research on the histopathology of ureteral strictures, which is detrimental to the management of ureteral strictures. In this study, three main types of ureteral stricture histopathology were identified: inflammatory cell infiltration, fibroplasia, and hyalinization. The variation in fibroblasts, inflammatory cells, and type I and III collagen among different pathological types has significant implications for further elucidation of the molecular mechanisms of ureteral strictures.

In pulmonary and liver fibrosis, the extracellular matrix produces fibroblasts that are activated into myofibroblasts. The accumulation of myofibroblasts leads to collagen deposition and disease progression (23), and inhibition of myofibroblast accumulation inhibits the progression of pulmonary and liver fibrosis (24). We therefore examined the changes in numbers of fibroblast in ureteral strictures. And we found that fibroblasts remained the most abundant in the fibroplasia types of ureteral strictures (Figure 2J), which led the process of cell proliferation and protein deposition (19, 20, 25), and we came up with the idea that fibroblasts may act strongly on the strictures of ureters, which may be an accumulative process.

Ureteral strictures are generally reported to have several etiologies such as stones, surgical injuries, malignancy, and trauma (26). And we have found that stones, injuries and even sutures left over from surgery can lead to ureteral strictures. In addition, stone-induced ureteral strictures were commonly seen in clinical practice. We counted and statistically analyzed the number of lymphocytes, fibroblasts, eosinophils, monocytes, foreign-body giant cells, and neutrophils in each of the three pathological types and three causes of diseases (Figures 2G,H,K–R). Among the three etiologies, lymphocyte, monocyte and neutrophil levels were significantly higher in strictures caused by stones and sutures than in those caused by injuries (Figures 2G,K,Q), reflecting a more severe inflammatory response in strictures caused by these two etiologies. The number of foreign-body giant cells, associated with foreign body reactions, was significantly higher in sutures, showing a stronger foreign body reaction, which reminds clinicians to use absorbable suture as thin as possible when performing ureteral suture surgery to avoid strictures caused by sutures. Interestingly, the number of foreign body giant cells around the stone was comparable to the number of foreign body giant cells in the injury and significantly lower than those around the suture (Figure 2O). Stones and sutures are made of different materials and located in different parts of the ureter. Sutures are mostly located in the ureteral wall, while stones embedded in the tube wall are rare. Therefore, in terms of pathological changes, the suture is mainly infiltrated with foreign body giant cells, while there are few such cells in stones. It can be seen that there is different mechanism of ureteral stenosis caused by stones and sutures.

Among the three pathological types, we observed significantly more lymphocytes (Figure 2H), eosinophils (Figure 2N), monocytes (Figure 2P) and neutrophils (Figure 2R) in the inflammatory cell infiltration type than in the hyalinization type. The number of fibroblasts was significantly higher in the fibroplasia type than in the other two types (Figure 2J). Therefore, we speculate that the inflammatory cell infiltration and fibroplasia types may belong to the early stages of fibrosis, where inflammatory cells activate fibroblasts by secreting cytokines or directly transform into fibroblasts, thereby increasing the number of fibroblasts and promoting disease progression.

As for the pathological type of inflammatory cell infiltration, we observed lower levels of type I and type III collagen (Figures 1G,J), and the inflammatory cells did not rank the same as collagen. Studies on tissue repair in embryos have shown that immature tissue can heal without scarring before the wound develops an inflammatory response to trauma, suggesting that inflammatory cell infiltration may be the cause of fibrosis (27). Therefore, we speculate that inflammatory cell infiltration might be the “start button” for the fibrosis, initiating an increase in collagen while stimulating the local fibroblasts.

In the present study, we found that the hyalinization type had the highest grade of hydronephrosis, while the inflammation type had the lowest grade of hydronephrosis, which may be associated with the longer obstruction history of hyalinization and fibroplasia compared with inflammation, with the longer course leading to more severe hydronephrosis and possibly a worse prognosis.

It has been mentioned that the increased degradation of type I collagen or increased deposition of type III collagen could be useful in combating excess rigidity of the interstitial matrix in tissues and that fibroblasts and inflammatory cells decrease along with scar formation (28). We wondered whether there was a similar manifestation in the duration of ureteral strictures. We found that in hyalinization, almost all types of cells that we observed tended to be less abundant, not only the inflammatory ones but also the fibroblasts (Figures 2H,J,N,R). In contrast, type I and type III collagen levels of hyalinization were the highest among the three pathological subtypes (Figures 1G,J). An inevitable consequence of tissue repair is fibrosis and scarring during the remodeling (29). We believe that hyalinization may be an advanced stage of the disease. We speculate that it may be that most areas of the ureteral stricture tissue are filled with extracellular matrix, therefore, the blood supply is reduced and the number of fibroblasts is decreased.

Type I collagen is a relatively hard and strong collagen that is responsible for the mechanical resistance of the tissues (30). Type III collagen, on the other hand, is less abundant and weaker than type I collagen (30). In our study, type I and type III collagen were both significantly elevated in patient samples with longer disease duration (Figures 1H,K), and the same phenomenon was observed in stricture vs. atresia (Figures 1I,L), possibly explaining the general feeling of firmness in the ureteral atresia. The amount of type I and type III collagen also suggests a pattern of fibrotic disease progression. It is possible that in the advanced hyalinization subtype, type I and type III collagen contents increase and fills the tissue, harden it and obstruct the lumen of the ureter, eventually leading to ureteral strictures or even atresia. This could explain the higher content of type I and III collagen in atresia than in stricture, as well as the higher content of type I and III collagen in those with a disease duration greater than 1 year than in those with less than 1 year. From a clinical perspective, we found that the longer the duration of ureteral stricture and ureteral atresia may contain more collagen fibers, which makes it difficult for patients with long obstruction history be cured by simple intraluminal expansion, and it may be a better choice to remove the damaged tissue for ureteral reconstruction.

However, there are limitations to our study, the sample size is small, and we had limited access to specimens as the primary treatment for ureteral strictures is the endoluminal ureteral technique. More potential pathological stenoses might have been identified with a larger sample size. Besides, further research is needed to elucidate the clinical role of three different pathological types of ureteral stricture.

## Conclusion

Three pathological types of benign ureteral strictures were identified: inflammatory cell infiltration, fibroplasia, and hyalinization. Changes in type I and III collagen, inflammatory cells, and fibroblasts in different pathological types may be related to the progression of ureteral strictures. Follow-up studies will confirm whether fibroblasts play an important role in the initiation of ureteral strictures.

## Data Availability Statement

The original contributions presented in this study are included in the article/Supplementary Material, further inquiries can be directed to the corresponding author/s.

## Ethics Statement

The studies involving human participants were reviewed and approved by the Ethics Committee of The First Affiliated Hospital of Chongqing Medical University. The patients/participants provided their written informed consent to participate in this study.

## Author Contributions

JT, ZY, and XY acquired the data. XL and ZY drafted the manuscript. YT and GQ contributed pathomorphological analysis. DY provided imaging data. FG and MY designed the study and revised the manuscript. All authors have made critical revisions to the manuscript.

## Conflict of Interest

The authors declare that the research was conducted in the absence of any commercial or financial relationships that could be construed as a potential conflict of interest.

## Publisher’s Note

All claims expressed in this article are solely those of the authors and do not necessarily represent those of their affiliated organizations, or those of the publisher, the editors and the reviewers. Any product that may be evaluated in this article, or claim that may be made by its manufacturer, is not guaranteed or endorsed by the publisher.
