# Supplementary material for: Main Pathological Changes of Benign Ureteral Strictures
Source: Front Med (Lausanne). 2022 Jul 7;9:916145. doi: 10.3389/fmed.2022.916145 (PMC9300898; doi:10.3389/fmed.2022.916145)
Supplement: Supplementary file 1 [file Table_1.DOCX]

Supplementary Material

**Supplementary Figure 1.** Characteristic imaging of ureteral stricture

(A): Ureteral atresia shown by retrograde urethrography. (B): Ureteral atresia shown by antegrade pyelography. (C): Ureteral stricture shown by retrograde urethrography. (D): Hydronephrosis shown by CT. The red arrow indicates the atresia, yellow arrow indicates the stricture.
