# Supplementary figures and images for: Main Pathological Changes of Benign Ureteral Strictures
Source: Front Med (Lausanne). 2022 Jul 7;9:916145. doi: 10.3389/fmed.2022.916145 (PMC9300898; doi:10.3389/fmed.2022.916145)

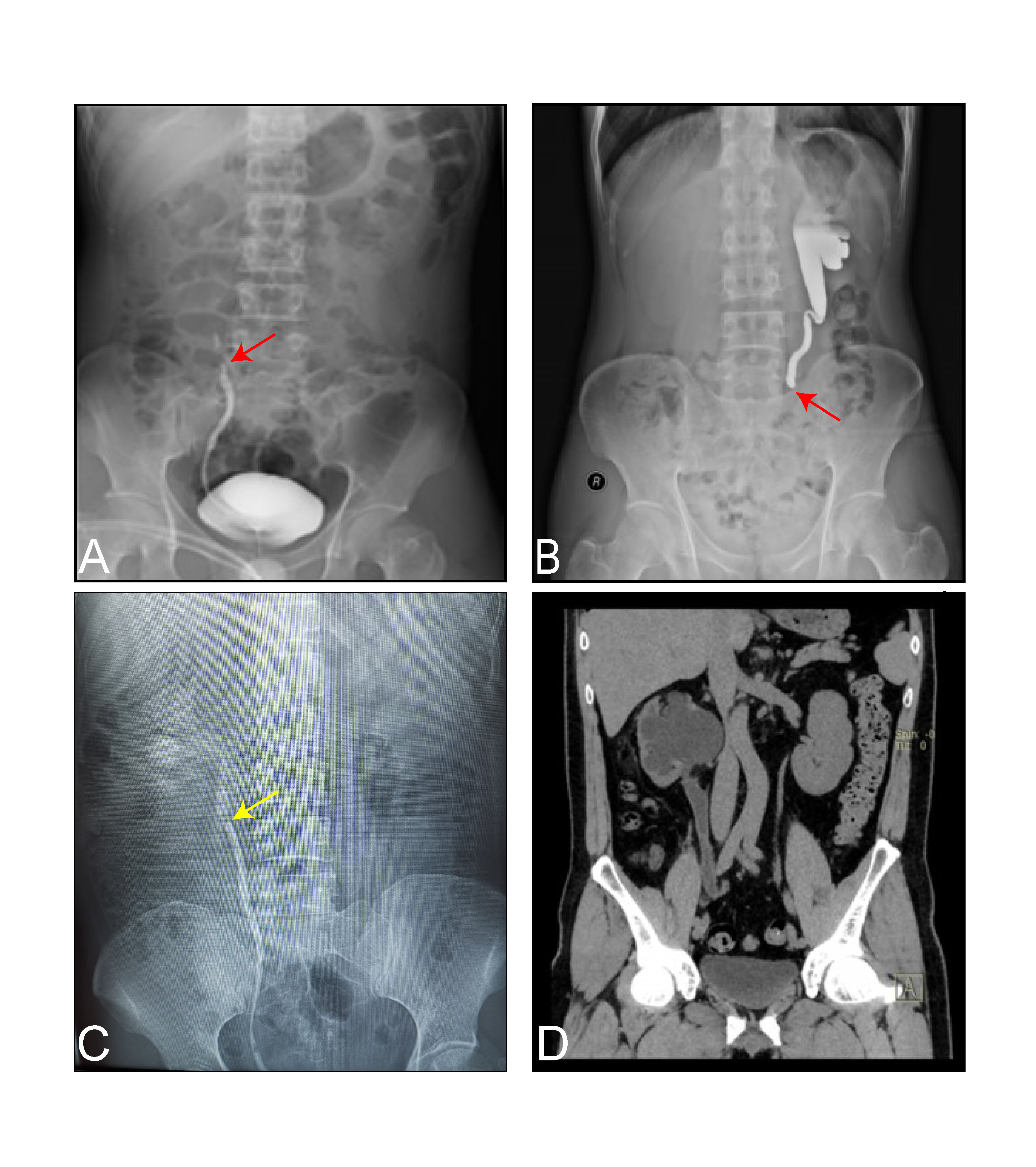

Supplement: Supplementary file 2 [file Image_1.JPEG]
